# A Scoping Review to Identify Community- and Societal-Level Strategies Evaluated from 2013 to 2023 for Their Potential Impact on Child Well-Being in the United States

**DOI:** 10.3390/children11091070

**Published:** 2024-08-31

**Authors:** Katie A. Ports, Whitney L. Rostad, Peter Coyne, Jadyn Dunning, Andrea E. Gonzalez, Adam Troy

**Affiliations:** American Institutes for Research, 1400 Crystal Drive, 10th Floor, Arlington, VA 22202-3289, USA; wrostad@air.org (W.L.R.); pcoyne@air.org (P.C.); jdunning@air.org (J.D.); dgonzalez@air.org (A.E.G.); atroy@air.org (A.T.)

**Keywords:** social determinants of health, child well-being, prevention, scoping review

## Abstract

There is increased recognition for solutions that address the social determinants of health (SDOHs)—the context in which families are raising children. Unfortunately, implementing solutions that address inequities in the SDOHs has proven to be difficult. Many child and family serving systems and communities do not know where to start or do not have the capacity to identify and implement upstream SDOH strategies. As such, we conducted a scoping review to assess the status of evidence connecting strategies that address the SDOHs and child well-being. A total of 29,079 records were identified using natural language processing with 341 records meeting inclusion criteria (e.g., outcomes focused on child well-being, interventions happening at a population level, and evaluations of prevention strategies in the United States). Records were coded, and the findings are presented by the SDOH domain, such as strategies that addressed economic stability (n = 94), education access and quality (n = 17), food security (n = 106), healthcare access and quality (n = 96), neighborhood and built environment (n = 7), and social and community context (n = 12). This review provides an overview of the associations between population-level SDOH strategies and the impact—good and bad—on child well-being and may be a useful resource for communities and practitioners when considering equitable solutions that promote thriving childhoods.

## 1. Introduction

The conditions in which children develop have profound implications for their lifelong health and well-being. The social determinants of health (SDOHs) are the conditions in the environments where people are born, live, learn, work, play, worship, and age that affect a wide range of health, functioning, and quality-of-life outcomes and risks [1]. Research has long highlighted the connection between SDOHs and exposure to adverse childhood experiences (ACEs) during the first 18 years of life [2]. ACEs include child abuse and neglect (CAN), as well as living in poverty, experiencing racism, and living in households with substance misuse [3]. There are strong associations between exposure to ACEs and well-being consequences, including changes in the physiological development of the nervous, endocrine, and immune systems; physical and mental health problems; engagement in risky health behaviors; limited life opportunities; and premature death [4,5,6,7,8,9,10].

Experiencing adversity in childhood is not inevitable and is widely recognized as a preventable public health problem [11]. However, conditions like poverty, racism, and historical trauma keep some children and families at greater risk of ACEs [12,13,14]. The disproportionate burden of adversity experienced by some children and families, primarily those of color, without citizenship, and living in low-income households, is increasingly understood as a major contributor to health inequities in the US [15]. And research consistently demonstrates that exposure to adversity is not randomly distributed but instead is disproportionately concentrated in historically disinvested, segregated communities that experience high rates of concentrated poverty, unemployment, family disruption, residential instability, and general geographic and social isolation because of systemic racism [16,17,18,19,20].

Racial disproportionality is particularly evident in systems that are meant to respond to children, youth, and families in need (e.g., child welfare and juvenile justice systems). For example, the child welfare system is responsible for responding to suspected and confirmed CAN cases. The overall rate of victimization was 8.1 per 1000 children in 2021, but 13.1 for Black children compared with 7.1 for White children [21]. Noticeable indicators of poverty (e.g., unstable housing, food insecurity, and inadequate clothing) often trigger reports to child abuse hotlines, and nationally, more than 60% of child welfare decisions are related to poverty [22]. Although all populations are at risk of experiencing adversity, families of color are more likely to encounter the child welfare system because of the structural and social conditions that perpetuate intergenerational poverty. As such, addressing the social and structural determinants that influence the conditions in which families are raising children can point to powerful levers of change that can sustain long-term prevention and health promotion.

The distribution of the SDOHs is often determined by socioeconomic and political contexts [13,14]. Attending to the distribution of the SDOHs provides important opportunities for efforts focused on child well-being to move beyond behavior change toward changing environmental conditions that keep some communities at higher risk. However, implementing solutions that address inequities in SDOHs has proven difficult [23,24]. Child-serving systems may not know where to start or do not have the capacity to identify and implement upstream SDOH strategies. Siloed health and human services systems and limited resources to operationalize SDOH frameworks into practical guidance have likely contributed to a lack of uptake among communities and practitioners to promote child well-being despite growing interest and recognized need. Efforts to identify, summarize, and implement innovative, evidence-based solutions can be slowed by a lack of human resources and the overwhelming number of publications available. To address this challenge, text analytics and natural language processing (NLP) tools can be used to automate and expedite a literature review of strategies that address SDOHs and focus on child well-being. These tools augment traditional literature review processes to allow for faster and more sophisticated categorizing, filtering, and searching of large sets of the peer-reviewed literature as well as the gray literature (e.g., reports and white papers). 

The purpose of this paper is to conduct a scoping study to assess the status of evidence connecting societal- and community-level SDOH strategies to child well-being from 2013 to 2023 using NLP tools. The rationale for choosing this period is that it covers the most recent decade of research on this topic, which has seen significant developments in theory, methods, and application. The 2013–2023 timeframe for the scoping review also encompasses the impact of the COVID-19 pandemic, which exposed and exacerbated existing health and social inequities in the US and has disproportionately affected children and families living in poverty, facing discrimination, or experiencing violence. Therefore, the scoping review can capture efforts to address the SDOHs before the pandemic, as well as emerging evidence on how it has influenced new efforts to address child well-being, and identify best practices and lessons learned for promoting resilience and recovery. Scoping reviews allow researchers to synthesize the existing literature in terms of volume, nature, and characteristics, particularly when the topic has not been extensively reviewed or is complex or heterogeneous in nature [25]. Given the multi-factorial nature of SDOHs and the myriad studies investigating child well-being, a scoping review has the potential to synthesize a broad body of research connecting SDOHs and child well-being, highlight gaps in the research, and offer insight into novel SDOH prevention and intervention strategies that address inequitable conditions that put children at risk. Importantly, this collection of research can highlight strategies that result in population-level change, which can maximize communities’ investments in prevention and move them beyond individual-level prevention strategies. 

## 2. Materials and Methods

We used PRISMA guidelines and other recommended protocols [25,26,27] to conduct a scoping review of records to identify strategies that address inequities in the social determinants of child well-being. See the PRISMA checklist (S1) in the Supplementary Materials. NLP tools were used to identify peer-reviewed articles, dissertations, theses, and governmental and nongovernmental publications published from 2013 to 2023 through a search of electronic databases: PubMed Central, Google Scholar, Elicit, ArXiV, and EBSCOhost. Because we were focused on strategies that address conditions and not individual behavior change, the model was trained to identify SDOH records that focused on original evaluations of population-level interventions or strategies (e.g., community, city, state, national) and well-being outcomes for children under 18 years. See Table 1 for search terms identified and refined by our team of subject matter experts. Once records were gathered, the NLP tool filtered the documents, categorized them by SDOH topics, and labeled the documents. While there are many SDOHs such as safe housing, accessible transportation, and racism, they generally fall into five domains [1]; economic stability, education access and quality, social and community context, healthcare access and quality, and neighborhood and built environment. We added a sixth SDOH domain to account for food and nutrition policies and programs, which were numerous and often cut across SDOH domains (e.g., economic security and social and community context).

A total of 29,079 records were identified using the search terms and parameters identified in Table 1. The NLP tools flagged 1548 articles for inclusion based on inclusion and exclusion criteria. Inclusion criteria included the following: (1) published between January 2013 and November 2023; (2) written in English; (3) conducted in the United States; (4) the record focused on population-level interventions or strategies; (5) the record was an evaluation; and (6) child well-being was an outcome. Exclusion criteria included the following: (1) the record was a review or commentary; (2) prevention or intervention focused exclusively on individual-level behavior change; or (3) the record focused on international work. Rigorous evaluation was not a criterion for inclusion, and not all prevention strategies identified have been rigorously evaluated. These titles and abstracts were then reviewed by two co-authors to determine if they truly met inclusion criteria. Duplicate records were removed (n = 177). Following the title and abstract review, 857 records were excluded for not meeting the inclusion criteria.

**Figure 1:**
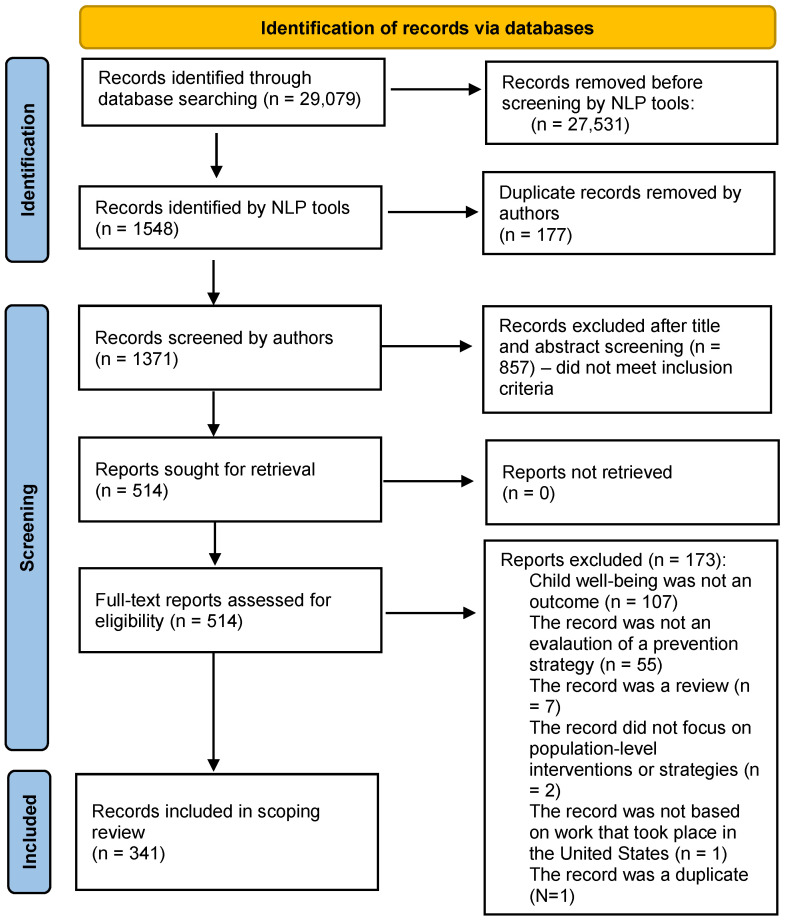
Prisma flow diagram of record inclusion selection. Note: natural language processing = NLP.

Research team members independently conducted a full-text review of 514 records to determine eligibility for inclusion with a 20% overlap for consensus agreement using the inclusion and exclusion criteria above. A review protocol was created based on the inclusion and exclusion criteria and stored in Airtable so that all members could work from a shared space. When disagreement occurred (<5%), the team met to discuss and resolve all discrepancies. Of the 514 full-text records reviewed, 173 were excluded: 107 did not include child well-being as an outcome, 55 were not an evaluation of a prevention strategy, 7 were reviews, 2 looked at individual change, 1 was a duplicate, and 1 focused on international work. After reviews, 341 records met the inclusion criteria for the scoping review (Figure 1). The 341 included records were coded for (1) publication type (e.g., peer-reviewed publication or government publication); (2) SDOH domain; (3) strategy or intervention; (4) study population demographics (e.g., age or race and ethnicity); (5) study design (e.g., program evaluation, randomized controlled trial, or comparison group presence); (6) setting where the strategy was implemented (e.g., community, city, or state); (7) outcome of interest; and (8) results, including outcomes of interest measured and methods for measuring the outcome.

## 3. Results

Across all records (n = 341), the outcomes pertaining to child well-being were vast. The most common indicators were healthcare access, utilization, and insurance coverage (n = 67), followed by food security or nutritious food consumption (n = 62), physical health (n = 45), birth and infant outcomes (e.g., low birthweight, infant mortality; N = 35), child welfare involvement, maltreatment, and/or violence (n = 40), education access, quality, or achievement (n = 27), mental health or behavioral and cognitive development (n = 22), unique or mixed indicators (e.g., poverty and education access, food security and physical health; n = 20), child poverty (n = 18), childcare (n = 3), and residential stability (n = 2).

Most records documented evaluations at the national level (n = 190), followed by individual states (n = 64), individual municipalities (n = 27), groups of states (n = 20), groups of municipalities (n = 10), groups of counties (n = 8), individual counties (n = 8), education sites (n = 5), unique (n = 2), mixed (n = 2), metropolitan area (n = 1), zip code (n = 1), and census region (n = 1). Additionally, a handful of articles did not specify the geographical setting of their study population (n = 5). Most records were peer-reviewed publications (n = 322) followed by working/white papers (n = 9), dissertations/theses (n = 8), and nongovernmental reports (n = 2). Below, we present findings for strategies that addressed a single SDOH domain, followed by findings from records that looked at the cumulative impact of multiple SDOH strategies. Findings for records that evaluated multiple strategies (e.g., Medicaid and tax credits), but did not look at the combined impact of multiple strategies, are reported in each of the respective SDOH domains. Accordingly, the number of records below does not reflect unique records, but the count for specific evaluations focused on each domain type. Strategies that addressed food security and nutrition (n = 106) were most common followed by healthcare access and quality (n = 96), economic stability (n = 94), education access and quality (n = 17), social and community context (n = 12), and neighborhood and built environment (n = 7). Twenty records considered the cumulative impact of multiple SDOH strategies on child well-being.

### 3.1. Economic Stability

In total, 94 records included evaluations specifically focused on economic stability strategies and their impact on child well-being. Many records focused on housing assistance/vouchers (n = 20) and tax credits (n = 19), which included 16 studies on the earned income tax credit (EITC) and 3 studies on the child tax credit (CTC); paid family leave (n = 14); and childcare subsidies (n = 13). Cash transfers (e.g., basic income, child allowances, and child development accounts) comprised 11 of the studies. An additional seven studies investigated welfare reform/Temporary Assistance for Needy Families (TANF), and five studies investigated minimum wage increases. There was one record for each of the following topics: youth employment, child support, cigarette tax, adoption subsidies, and tax policies that incentivize marriage.

#### 3.1.1. Housing Assistance

Housing assistance programs had mixed findings and were influenced by the type of housing assistance, the demographics of the populations served, and neighborhood characteristics. For example, project-based housing was associated with increased exposure to neighborhood disadvantage [28,29,30], did not improve or harm child health [31] nor high school graduation rates [29], but did result in improvements in residential stability [29,32] and mental health outcomes [33]. Whereas housing vouchers led to reductions in disadvantage for Black and Latino children [28], reductions in residential crowding, and improvements in high school graduation [29], they had no impact on mental health [33], academic achievement, and health outcomes [34]. Children in families receiving housing support were more likely to live near lower-performing and higher-poverty schools than children living in poverty without housing assistance [29,35,36]. Rental assistance, in general, was associated with a reduction in emergency department use among children with asthma [37] and reductions in school absenteeism [38]. And long-term rent subsidies reduced homelessness, food insecurity, school stability, absenteeism, and behavior problems [39].

The results indicated that the Family Unification Program (FUP), which offers housing support to families involved with the child welfare system, was associated with children’s access to reading and home quality [40], increased housing stability [40,41], decreased overcrowding [40], and foster care placement [42]. Nonetheless, the FUP and similar programs failed to significantly reduce system-wide rates of family separation [43,44], CAN [45], and housing affordability and neighborhood quality [40]. However, when housing assistance programs prioritized families experiencing homelessness, CAN rates declined [46] and stability improved [47], especially when housing was near schools [48].

#### 3.1.2. Tax Credits

The generosity of tax credits was found to consistently improve outcomes for children, with greater child well-being benefits associated with the generosity of the tax credits, including refundability. For example, the refundable child tax credit was associated with reductions in child poverty [49] and the rates of CAN [50] and fewer childhood injuries requiring medical attention and behavior problems [51]. Additionally, EITC generosity was associated with declines in child poverty in the state of Oregon [52], food insecurity [53], behavioral problems [54], CAN reports [55], foster care entry [56,57], and improved birth outcomes [58] with largest impacts seen among Black mothers and infants [59,60]. The EITC was also associated with improvements in overall health [61] and birth outcomes [62,63], especially for infants with Black mothers [64,65]. However, Qian found that the EITC was not associated with birth outcomes once place-based effects were controlled [66]. The EITC had mixed effects on childhood adiposity where children who were obese were negatively affected, but underweight children saw improvements [67].

#### 3.1.3. Paid Family Leave

Paid family leave (PFL) was associated with reductions in infant hospitalizations [68,69], pediatric abusive head trauma [70], the risk of health problems [71,72], and post-neonatal mortality rates [73]. In addition, PFL policies improved immunization rates [74,75], mother–child interactions and attachment security [76], social and emotional skills [72,77], high school graduation rates [78], and breastfeeding initiation for Black women and their infants [79]. While behavioral problems among children of younger parents were reduced among families who had access to PFL, Black children and children of high-income and older parents experienced increased behavioral problems [80]. In San Francisco, PFL did not affect birth outcomes among marginalized groups, who remain at risk of poor outcomes because of structural racism [81].

#### 3.1.4. Childcare Subsidies

Policies that offset the costs of childcare through subsidies and tax credits reduce child poverty rates [82]. The Child Care Development Fund (CCDF) is the primary funding source for childcare subsidies, which had mixed findings on child outcomes. While the administrative burden of the CCDF was inversely associated with children’s continuous enrollment in childcare [83], subsidy utilization was associated with higher perceived quality of care [84,85], positive child development [86], and reduced absenteeism and improved academic outcomes for those who received care from a licensed provider [87]. However, childcare subsidies were not independently related to children’s behavior problems or vocabulary in the Fragile Families Study [88] and negatively impacted academic outcomes among children with special needs [89] and among children attending community-based centers [90]. In general, the presence of subsidies was not associated with CAN rates [91]; however, when subsidy generosity and access increased, states saw reductions in abuse and neglect rates [92,93], and children experienced fewer home removals in states with CCDF policies that made accommodations for children in CPS and foster care [94].

#### 3.1.5. Cash Transfer Programs

Cash transfer programs demonstrated significant improvements in child well-being outcomes. For example, Alaska’s Permanent Fund Dividend, a universal basic income program, was associated with reductions in Alaska Native child poverty [95]. Indiana’s Wabash County Promise Scholarships program, which combines children’s savings accounts with scholarships, resulted in improvements in academic outcomes, especially for students from low-income households [96]. SEED for Oklahoma Kids, a statewide child development account program, demonstrated positive social–emotional development outcomes for children, especially for those from low-income households [97,98,99], as well as improved financial outcomes (i.e., students had greater savings for college) [99]. Opportunity New York City (NYC)—Family Rewards, a holistic conditional cash transfer program, reduced aggression and substance use among adolescents [100] as well as poverty but did not impact child education and health outcomes [101]. Riccio and Miller [102] also found that Opportunity NYC improved food and financial security, preventive dental care, graduation rates, and other positive school outcomes. Maine’s Harold Alfond College Challenge, a children’s savings account program, was associated with increased savings for college [103]. And finally, simulations of a universal child allowance of USD 250 a month showed that child poverty and deep poverty would significantly decline [104] and that the social benefit of a child allowance (e.g., children’s future earnings, tax contribution, health, etc.) was far greater than the cost per year [105].

#### 3.1.6. Welfare Reform

The relationship between child well-being and welfare reform/TANF is mixed and influenced by state-level implementation and the racial/ethnic composition of the population served. Increases in TANF benefits resulted in declines in physical abuse [106], neglect [107], improved family daily routine [108], and academic outcomes [108,109]. However, TANF was not found to enhance cognitive stimulation and family interactions [108]. In addition, welfare reform was associated with worse health outcomes and reduced healthcare utilization among children [110], greater perpetration of bullying [111,112], and states that implement harsh waivers which are more likely to see poorer ratings of child health and increased hospitalizations [113].

#### 3.1.7. Minimum Wage

Increasing the minimum wage was associated with positive outcomes for children, including fewer CAN reports [114,115], reductions in violent behavior among children [116], and fewer externalizing behaviors [117]. However, minimum wage did not demonstrate significant effects on birth outcomes once place effects were controlled [68].

#### 3.1.8. Other Strategies

Five other strategies were identified that targeted economic security and child well-being. Child support policies, youth employment programs, and cigarette tax resulted in positive outcomes for children. First, enforcement of child support policies was associated with declines in child death rates [118]. Second, Cleveland’s summer youth employment programs demonstrated declines in juvenile offense filings and improved school attendance and graduation rates [119]. Finally, in-utero exposure to a cigarette tax hike decreased sick days from school and the likelihood of having two or more doctor visits in the past 12 months [60]. The two other policies were not as effective. Adoption subsidies did not improve foster care adoption rates [120]. And although tax policies that incentivize marriage were associated with declines in child poverty, they increased deep poverty for single-mother families [121].

### 3.2. Healthcare Access and Quality

A total of 96 records focused specifically on healthcare strategies and their impact on child well-being, the majority of which focused on public insurance benefits (i.e., Medicaid and the Children’s Health Insurance Program [CHIP]; n = 82) followed by maternal and infant healthcare strategies (n = 6), mental healthcare (n = 3), and family planning (n = 2). School health centers, vaccination programs, and in vitro fertilization (IVF) insurance were also included in one record each.

#### 3.2.1. Public Health Insurance

Recognizing the important role insurance plays in healthcare access, the federal government has implemented several policies to support increased enrollment in public health insurance policies. Among those are the Affordable Care Act (ACA), which expanded Medicaid, and the Children’s Health Insurance Program Reauthorization Act (CHIPRA), which expanded CHIP. Notably, the ACA expansion resulted in increased insurance coverage for children and youth [122,123,124,125], including pediatric cancer patients [126], and increased well-child visits for Chinese youth [124]. The ACA also mandated coverage of lactation services, which resulted in increased breastfeeding, especially among Black and American Indian/Alaskan Native Mothers [127]. CHIPRA was similarly associated with decreases in uninsurance rates [128] and a substantial drop in unmet health needs [129], including among immigrant children [129,130]. While the adoption of public health insurance expansion (i.e., CHIPRA and ACA) was not associated with enrollment disparities, such inequities have decreased over time in all [131].

##### Medicaid

Access to and generosity of Medicaid benefits was associated with increased insurance coverage [132,133,134], access to care for children with disabilities [135], pediatric cancer survival [136], high school completion for Hispanic and White students [137], generational economic mobility [138], higher blood lead levels [139], and victimization and perpetration of bullying [111,112]. Medicaid access and generosity were also associated with reductions in cases of CAN [140,141] but not child sexual abuse [142], low birthweight and preterm birth [143], infant and child mortality among children of color [144,145], child poverty [146], and the socioeconomic and White–Black achievement gap for math [147]. However, Medicaid increased reliance on acute care settings among adolescents versus preventive care [148].

Additional Medicaid policies have had varied impacts on child well-being outcomes. For example, Pay for Performance was not associated with the completion of the vaccine series [149], and enrollment rule easing was not associated with birth outcomes [150]. But voluntary and mandatory nonpayment policies reduced the rate of early-term births and low birthweight among women who smoked preconception [151]. Coordinated Healthcare for Complex Kids was not associated with changes in medical expenditures [152] nor school absenteeism [153] but did show some improvements in healthcare utilization and prescriptions [153]. Children with capitated plans had greater odds of healthcare utilization and lower odds of emergency department visits compared to those in fee-for-service plans [154]. And children with continuous Medicaid eligibility had improved insurance coverage, access to medical care, and better health ratings [155]. Home- and Community-Based Services Medicaid waivers were associated with increased insurance coverage and reduced unmet health needs among children with serious emotional disturbance [156] and children with autism spectrum disorder [157]. Medicaid Managed Care increased healthcare utilization among low birthweight infants [158], the diagnosis of ADHD and asthma, and emergency room visits [159], but did not reduce outpatient utilization among foster children as much as other children with Medicaid benefits [160]. Moving to managed care models was also associated with deterioration in birth outcomes in Pennsylvania [161], and Medicaid Accountable Care Organizations were not associated with infant health outcomes [162].

While Medicaid generally resulted in improved healthcare access, evidence on the quality of services was mixed. For example, children with congenital cardiac operations on Medicaid had increased mortality, readmissions, care fragmentation, and costs compared to those with private insurance [163]. Families participating in Louisiana’s Family Opportunity Act Medicaid Buy-In Program were less likely to receive help with care coordination [164]. And TexKat, the largest Hurricane Katrina Medicaid Emergency Waiver, prevented extreme utilization disruptions, but there were some indications that children received inadequate care [165]. Furthermore, compared to children with public or no health insurance, children with private insurance were more physically active [166] and more likely to have had a routine checkup in the previous twelve months and less out-of-pocket spending [167], but had similar dental care utilization [168].

##### Medicaid Dental Care

Policies that provided dental coverage under Medicaid had mixed findings. Some records reported increased positive oral health outcomes [169], oral healthcare utilization [170,171], and reduced emergency department visits for non-traumatic dental conditions [172]. Other records found that such policies did not increase the use of preventative measures among elementary students in Texas [173], nor nationally [172]. Adult dental coverage was associated with oral healthcare utilization among children [174,175], but other studies found no impact on parent–child dental use with Medicaid expansion [176]. However, North Carolina’s Medicaid preventive dentistry program, Into the Mouths of Babes, reduced dental caries among targeted vulnerable children, which helped reduce oral health disparities among preschool-aged children [177].

##### CHIP

In addition to Medicaid, CHIP provides public health coverage to eligible children through both Medicaid and separate CHIP programs using state and federal funds leading to variations in implementation across states. These variations account for variations in child coverage, especially for poor and foreign-born children who experience the greatest inequities in coverage [178]. CHIP access and generosity have resulted in increased insurance coverage [179,180] and preventive healthcare utilization [181]. Similarly, increasing CHIP premiums nullified the effects of CHIP expansions [182] and increased private insurance rates for children [183].

##### Medicaid and CHIP

Policies that improved the generosity of public health insurance programs or that reduced administrative burdens (e.g., express lane and reducing copays) were associated with Medicaid and CHIP enrollment among children [184,185], including among children with foreign-born parents [186,187]; resulted in improvements in child health outcomes [188,189], third-grade reading scores [190], and mental health outcomes for teenagers [191]; and were associated with declines in CAN rates [92]. Furthermore, disparate public health insurance coverage among siblings resulted in poorer health outcomes for children who did not have access to CHIP or Medicaid [192,193]. And when parents lose health insurance coverage, children also risk losing coverage even when public coverage for children expands [194].

##### Prenatal Care

Expanding prenatal access to public health insurance improved healthcare utilization and infant and maternal health outcomes for unauthorized immigrant women [195] and Black mothers [196]. However, expanding prenatal care access to foreign-born Latinas through CHIP did not change birth-related outcomes [197,198]. Prenatal care also had positive spillover effects on older siblings’ healthcare utilization if they were 3 to 4 years older than the infant [199].

#### 3.2.2. Maternal and Infant Health Programs

In some cases, home visiting is delivered by healthcare professionals or families receive maternal and infant care support through partnership programs with the healthcare system. These programs aim to improve the access and quality of healthcare for families to improve infant and maternal health outcomes. For example, the Child Health Investment Partnership of Roanoke Valley, Virginia improved dental care and oral health [200]. In Michigan, American Indian births in medically underserved counties had decreased odds of low birthweight and inadequate prenatal care when families participated in the Healthy Start program [201]. The Healthy Start program improved infant health outcomes in St. Louis [202], but not in South Carolina [203]. Home visits by a nurse were associated with improvement in language and cognition skills [204], and the Michigan Maternal and Infant Health Program improved child healthcare utilization [205].

#### 3.2.3. Mental Health

Mental health is critical to child well-being, and yet parity of mental health services remains a challenge. Policies and programs have been implemented to address this challenge. For example, the Mental Health Parity and Addiction Equity Act led to declines in out-of-pocket spending on child mental health and substance use disorder treatment [206], and improved healthcare utilization [207]. The Children’s Full-Service Partnership (FSP) program, an intensive in-home mental health service program for children aged 0–15 and their families, resulted in higher mental health emergency services rates initially, reflecting greater severity, but mental health emergency services trajectory declined more rapidly than those not in FSP [208].

#### 3.2.4. Other

Vaccination programs and school-based healthcare offer alternative strategies to addressing healthcare access and quality issues that impact child well-being. For example, implementation of elementary school-based health centers in Georgia increased the receipt of preventive care, with reductions in racial and ethnic disparities [209]. And while Vaccines for Children Programs are helpful, they do not seem to improve influenza vaccination rates among children [210]. Children of color born after family planning programs that provided birth control and education began in the 1960s and reduced child poverty [211]. Access to publicly funded family planning in California was associated with fewer teen pregnancies [212]. And health insurance, in general, was associated with declines in risks for teen pregnancy [213]. Finally, IVF insurance coverage mandates were associated with significant increases in adoption rates [120].

### 3.3. Education Access and Quality

We identified 17 records focused on education access and quality. The records focused on Head Start (n = 8) and other early education programs (n = 9). Access to early education has long been recognized as an essential determinant of children’s educational trajectories and their subsequent health and well-being. For example, in the 1940s the government funded universal preschool under the WWII Lanham Act Nursery School, and despite limited knowledge of child development at the time, demonstrated positive impacts on boys’ academic outcomes through high school [214]. Since then, the federal government and states have supported enriched early education opportunities including Head Start and school voucher programs. In national evaluations of Head Start, findings have demonstrated positive effects on economic security during the Great Recession [215], enhanced academic skills for children aged 5–6 years with multiple disabilities [216], improved social–emotional skills [217], and improved weight for those who started with an unhealthy weight [218]; however, effects on body mass index (BMI) were not significant for the general population [219]. In Tulsa, Oklahoma, Head Start improved academic achievement and reduced absenteeism, especially among Hispanic and female students [220]. While promising, other studies found that students in Tulsa who attended pre-K, but not Head Start, missed less school, were less likely to be retained in grades, and more likely to take advanced courses, but did not have higher test scores or grades compared to Head Start students, and findings were more positive for Hispanic, Black, and American Indian Students [221]. In terms of physical health benefits, access to early education through universal pre-k programs accelerated the rate at which NYC children were identified with and treated for various health conditions [222], and improved oral healthcare in North Carolina [223,224].

School voucher programs resulted in positive academic outcomes [225] and improved high school graduation rates [226]. In addition to the positive benefits of participating in early education opportunities, school funding was associated with child well-being. For example, participation in Head Start followed by enrollment in better-funded schools increased high school graduation rates [227], and programming funded by Title I was associated with a reduction in academic gaps by race and socioeconomic status (SES) [228]. Parent and child preschool participation in Chicago’s child–parent centers was associated with significantly lower rates of adverse health outcomes [229] and improvements in school readiness and academic outcomes [230].

### 3.4. Food Policies/Programs

Interventions focused on the impact of food and nutrition policies and programs on child well-being were the most common records (n = 106). Food and nutrition strategies seek to offset the cost of nutritious food, help address food insecurity, promote nutrition and healthy diets as part of good health and well-being, and often address food deserts in communities. Because these prevention strategies cut across SDOH domains and because of the volume of records, they represent their own subcategory. Most records looked at federal- and state-funded food subsidy programs (n = 96), but some included novel food and nutrition programs in communities (n = 7), as well as lesser-known nutrition programs like the United States Department of Agriculture’s (USDA’s) summer food service program (n = 1) and the Child and Adult Care Food Program (CACFP; n = 2). The CACFP is a federal program providing reimbursements for nutritious meals and snacks to children and adults enrolled in care centers.

#### 3.4.1. Supplemental Nutrition Assistance Program (SNAP)

SNAP benefits had positive associations with academic achievement [231,232], developmental outcomes [233,234], and physical health outcomes [234,235,236,237,238], especially among Hispanic children [239], diet and nutrition [240,241], and healthcare utilization [238,242,243,244,245]. SNAP benefits also reduced rates of CAN [246,247], school absenteeism [239,243], and food insecurity [234,238,248,249,250,251,252,253,254,255,256,257]. Similarly, losing access to SNAP or declines in benefits were associated with increases in poor health [248], school absenteeism, and decreased preventive healthcare utilization [258], especially among Black and Hispanic families [242].

Conversely, other studies showed that SNAP benefits did not significantly improve food security for children [259,260,261,262], physical health [263,264,265], or healthcare access [266]. In addition, SNAP was associated with an increased likelihood of illnesses, such as anemia and anaphylaxis [267,268], behavioral problems [244,269], social and emotional challenges [270], and greater victimization and perpetration of bullying [111,112]. And although diet was improved, children did not tend to meet dietary recommendations for healthy eating [271,272,273], especially towards the end of the month [274]. Similarly, students’ academic performance was affected by the recency of the benefit, peaking at week three [275]. Collectively, these findings suggest that SNAP has beneficial impacts on children, but benefits may not fully satisfy children’s nutritional needs, especially towards the end of the month when benefits have likely been used.

#### 3.4.2. The Special Supplemental Nutrition Program for Women, Infants, and Children (WIC)

Like the SNAP records, those evaluating WIC demonstrated a mixed impact on child well-being. WIC benefits were positively associated with food security [251,255,276,277], diet and nutrition [251,276,278,279,280,281,282,283,284,285], healthcare utilization [286], physical health outcomes [286,287,288,289,290,291,292,293,294,295,296], homework routines and consistent bedtimes [295], cognitive development [233,296], and oral [297] and mental [298] health. Outcomes varied by age, race, ethnicity, and poverty with children of color experiencing some greater impacts on well-being outcomes [283,296]. Conversely, other studies showed that WIC benefits did not improve food security for children during the COVID-19 pandemic [260], had no impact on breastfeeding [299], resulted in greater perpetration of bullying [111,112], were associated with a risk of higher blood lead levels [139], and generally, did not improve physical and mental health outcomes for children [264,300,301]. Indeed, long-term use was associated with higher obesity odds nationally [276].

#### 3.4.3. WIC and SNAP

The summer electronic benefits for children improved access to SNAP and WIC benefits [302] resulting in reduced food insecurity [303] and improved diet and nutrition [304,305]. The results suggest that SNAP and WIC enrollment is positively associated with diet and nutrition [251,306,307] with greater impacts experienced by populations at the greatest risk of adverse outcomes. For example, in a population of low-income, minority families with high-risk infants, enrollment in food assistance programs was associated with positive health and cognitive outcomes [308].

#### 3.4.4. School Meal Programs

Free and reduced meals are available to students across the country through various programs and policies. Findings from these strategies are mixed, often demonstrating some improvements, but not fully meeting the needs of children and families. For example, the National School Lunch Program (NSLP) reduced food insecurity [309,310,311]; however, the impact on physical health was varied. Nationally, lunch programs were not associated with fifth grade BMI [264], but were for White, Black, and Indigenous high school students [312]. Participation in breakfast programs was associated with improved behavior and academic outcomes [313]. Universal free meals at school were associated with increased readiness to learn and improved social climate [314]. However, studies showed that school meal programs did not improve food security for children nationally [259], nor in the Rio Grande Valley in Texas [315], and resulted in greater victimization and perpetration of bullying [111,112].

Efforts to expand reach and access have resulted in improved child well-being. Under the Community Eligibility Program, expansions to the NSLP and the School Breakfast Program reduced food insecurity [255], increased participation in the program [316,317], improved standardized test scores among Hispanic and White students [316,318], increased attendance, and among low-income families, decreased the probability of being overweight [316]. The Healthy, Hunger-Free Kids Act (HHFKA) of 2010 was associated with reductions in obesity for children in poverty [319,320] and improvements in diet and nutrition [321]. However, the HHFKA was also positively associated with high school obesity [322], and in Nevada, did not reduce child food insecurity [323].

#### 3.4.5. Other

We identified 10 records that included other food policies and programs meant to complement the benefits provided by SNAP, WIC, and free or reduced school meals. Nationally, the summer food service program increased geographic accessibility and reduced food insecurity [324], and the CACFP reduced food insecurity [325] and improved diet and healthy weight [326]. Meals on Wheels programs resulted in reductions in child food insecurity [327,328], but families still experienced severe hunger [328]. In Oklahoma, the packed-promise food box delivery program resulted in improved diet and nutrition in the Chickasaw nation [329]. Weekend backpack programs also reduced food insecurity [310] and improved school attendance, but only on Fridays when backpacks were distributed [330]. A food intervention based in a primary care setting showed improvements in healthy weight [331], and a kids-focused farmers’ market intervention in West Virginia resulted in improved diet and knowledge of fruits and vegetables [332].

### 3.5. Neighborhood and Built Environment

Neighborhoods and built environments contribute to the physical environment in which children live, learn, and play. Our review uncovered seven records focused on strategies that addressed the neighborhood and built environment to improve child well-being. Benefits were most notable for Black and Hispanic families. For example, a simulation of an NYC transportation and climate initiative found that various caps on on-road carbon emissions would reduce asthma and other respiratory illnesses and improve birth outcomes, and that Black and Hispanic children and those living in poverty would see the greatest improvements [333]. However, NYC’s introduction of a smoke-free housing policy was not associated with a decrease in healthcare encounters [334]. The empowerment zone program—a federal program that gave sizeable grants and tax breaks to certain high-poverty census tracts in selected cities—demonstrated differential impacts on birth outcomes by race and ethnicity with Black and Hispanic families experiencing benefits whereas White families did not [335]. The number of low-income housing units funded through the Low-Income Housing Tax Credit (LIHTC), which scores and funds housing development based on neighborhood factors, was associated with declines in CAN [336,337]. However, housing developments funded by the LIHTC zoned near racially and economically isolated schools were associated with especially high levels of economic and racial isolation for Black and Latinx students [30]. Children in San Francisco who lived in non-redeveloped public housing were more likely than those in redeveloped public housing or those not in public housing to experience repeated urgent care hospital visits [338]. Although housing is critical, where housing is placed plays a key role in whether and how children benefit.

### 3.6. Social and Community Context

Children’s relationships and interactions with family, friends, and community members can have a major impact on their well-being. We identified 12 records that focused on social and community context strategies, most of which focused on immigration policies (n = 8). The records that focused on the impact of restrictive immigration policies found decreases in insurance coverage [339,340,341,342] and food security [343,344], whereas the Deferred Action for Childhood Arrivals (DACA) program increased birthweights, high school attendance [345], and graduation rates among Hispanic children (Kuka et al., 2020; Torres et al., 2022) [345,346].

Additional records looked at other programs and policies that impact social and community interactions. For example, one record looked at cumulative federal and state funding of healthy marriage initiatives and found that these reduced the percentage of children living in or near poverty [347]. However, as noted by Ortigueir and Siassi [121], these types of policies can result in increases in deep poverty for single-mother families. Another record looked at the Child Access Prevention (CAP) gun laws and found lower rates of firearm mortality, particularly for non-Hispanic Black youth [348]. And home visiting was associated with improved developmental outcomes [233] and fewer substantiated CAN cases, but more unsubstantiated reports [349].

### 3.7. The Cumulative Effect of Prevention Strategies across Multiple SDOH Domains

While several records included multiple prevention strategies that cut across several SDOH domains, few considered their cumulative impact. As such, findings pertaining to one strategy are included under the specific domains in the previous sections. Here we report only the cumulative impact of prevention strategies across SDOH domains (n = 20). For example, states’ total spending across multiple public benefit programs was found to be inversely associated with CAN [350]. Similarly, TANF, SNAP, housing subsidies, and refundable tax credits combined keep roughly 2.38 million children from poverty but leave 1.17 million children behind [351]. Higher state-level generosity and take-up of SNAP and TANF significantly reduce extreme child poverty [352,353]. And compared to other safety net policies, the EITC had the largest impact on reducing racial inequities in birth outcomes [59]. In one simulation, findings suggested that adopting the most generous EITC policy would have the largest impact on child poverty, followed by SNAP, TANF, and lastly state CTCs, and that if all states were as generous or inclusive as the most generous or inclusive state in all four policies, 5.5 million children would be lifted out of poverty [354]. Similarly, health and non-health benefits to low-income families with children significantly reduced the poverty gap; however, Medicaid alone had the largest impact on child poverty than all non-health benefits combined [355], suggesting that EITC and Medicaid can make significant contributions to child well-being if implemented in ways that expand access and generosity to families in need. However, duration in poverty-related programs (i.e., TANF and Medicaid) was associated with an increased risk of CAN reports across races/ethnicities [356], likely due to increased surveillance of participating families. Importantly, public assistance programs can have differential benefits to individuals within a household that studies are often unable to tease out; however, one record found that combining school finance consolidation policies with public housing programs could improve child well-being without hurting other household members’ welfare [357].

Several records (n = 6) examined programs that move families out of low-poverty neighborhoods and cut across economic security (housing support) and neighborhood and built environments (neighborhoods with more resources) for their impact on child well-being. For example, children who participated in Baltimore’s housing mobility program experienced significant improvements in asthma symptom days and exacerbations [358]. The Moving to Opportunity project similarly demonstrated that participating families experienced higher neighborhood-level child opportunity [359,360], improved mental health outcomes, and better general health among children who spent more time in lower-poverty neighborhoods [360], and had lower rates of hospitalization and yearly inpatient spending [361], but had no impact on lifetime emergency room visits [362].

Another subset included the provision of wraparound services to children and families at risk of child welfare system involvement (n = 3) or embedding services within the child welfare system (n = 2). While these interventions are not primary prevention, they may be key intervention strategies that address the conditions of children and families at risk of violence. Unfortunately, when services are tied to child welfare, it appears that the impact on children is mixed. For example, programs that provided wraparound services for families involved with the child welfare system, including housing, health services, and job support, did not reduce CAN rates [349,363,364] but did improve behavioral and executive functioning outcomes [363]. Relying on either work, or a combination of work and welfare benefits (e.g., TANF and WIC), reduces the likelihood of system involvement compared to parents who rely on welfare benefits in the absence of work [365]. And finally, integrating TANF benefits into child welfare system involvement was associated with subsequent year, but not second year, increases in rates of substantiated CAN overall and neglect specifically [366].

## 4. Discussion

SDOHs have a major impact on children’s health, well-being, and quality of life. When not addressed, they contribute to wide health disparities and inequities [2]. For example, a lack of access to education, good-paying jobs, and wealth-building opportunities can perpetuate economic disparities, affecting generations. Addressing the inequitable distribution of SDOHs has posed challenges for communities; however, this review highlighted hundreds of studies that have demonstrated positive effects on child well-being through efforts that address SDOHs. Many records, including those investigating EITC, Medicaid, and caps on on-road carbon emissions, demonstrated decreases in gaps by race/ethnicity and SES and show promise for a better, more equitable childhood [64,65,147,333]. The mixed findings across strategies reflect differences in how various policies and programs are implemented and highlight practices that can result in harm and that widen inequities. Specifically, policies and programs that increase administrative burdens, lessen generosity, and support the concentration of poverty tend to negatively impact children’s well-being [28,30,32,53]. As such, it is not just whether a policy or program exists, but how it is implemented and who it reaches. Unfortunately, many records evaluating the effects of SDOH strategies did not consider for whom the policies and programs benefitted, and there remains a need in the field to not just look at impacts broadly but to also consider unintended consequences as well as who benefits and why.

This review highlighted gaps in the evidence. Notably, evidence for population-level impact on child well-being is lacking for social and community conditions, neighborhood and built environments, and education access and quality, whereas studies on public insurance and food subsidies were most prevalent. Importantly, policies and programs do not exist in isolation and often represent a complex interplay of factors that cut across many domains of SDOHs and outcomes, and yet, only 20 studies looked at the interplay and cumulative impact of multiple strategies. Many of these studies demonstrated positive, significant, and population-level effects on child well-being highlighting how strategies work together to change the conditions for children [351,355]. However, many studies also noted the need to do more to reach the most vulnerable populations [352]. As such, more work is needed to better understand the constellation of policies and programs that impact the lives of children, families, and communities.

## 5. Limitations

This scoping review is not without limitations. While we endeavored to capture all records, it is possible we missed some, especially those that are not published in traditional sources. We may have missed key records published before or after our selected period or records that were published in alternative databases. In addition, we excluded international records and those that were not published in English that may present additional innovative ideas that promote child well-being and that have not been considered in the context of the United States. We also excluded records that did not directly impact children. As such, many policies that benefit parental outcomes—key risk and protective factors for child well-being—were not included. Importantly, we did not consider the rigor of records, and as such, one should not assume the records demonstrate rigorous findings. We also excluded strategies that focused on individual-level impact. It is likely that SDOH strategies improve individual-level outcomes; however, we were focused on the evidence that impacted outcomes at a population level (e.g., county-level graduation rates or school district food insecurity). And finally, many strategies fit into multiple SDOH domains. For example, PFL was included under economic security. While PFL is intended to offset costs associated with taking time off to support family, it is also intended to promote parental bonding with new children, which could also fall into social and community contexts. Our team endeavored to categorize records based on the primary intent of a prevention strategy (not the outcome studied); however, we recognize the complexity of these interventions and that they may fit across multiple domains.

## 6. Conclusions

This review provides a comprehensive overview of the associations between population-level SDOH strategies and the impact—good and bad—on child well-being. Communities, practitioners, and policymakers have many options when it comes to promoting equitable and healthy childhoods; however, additional research is needed to better understand how strategies work across domains and work together to have a cumulative impact on safe, supportive, and nurturing environments. Indeed, for all children to thrive, we need a constellation of policies and programs that address SDOHs and provide them the opportunity to do better than previous generations.

## Figures and Tables

**Table 1:** Summary of search terms and parameters used to identify articles reporting on association between strategies that address social determinants of health (SDOHs) and child well-being.

Terms related to SDOHs	((policy OR program OR law OR place-based initiative OR partnership OR system OR community OR state OR population OR city OR county OR interrupted time series OR fixed-effects model OR QED OR quasi-experimental design OR RCT OR randomized control) OR (access to education OR high-quality education) OR (access to nutritious food OR nutrition) OR (accountable care organization) OR (administrative burden for benefit) OR (after school program OR youth program) OR (alternative payment model) OR (block grants OR community grants) OR (career education OR technical education) OR (child allowance) OR (child savings account OR baby savings account) OR (childcare subsidy) OR (child tax credit) OR AND (grant administration) OR (Head Start OR universal pre-k OR pre-k OR preschool OR pre-kindergarten) OR (health education) OR (health insurance OR Medicaid) OR (healthcare provider OR healthcare access OR healthcare availability) OR AND (earned income tax credit) OR (environmental justice) OR (family leave OR maternal leave OR paternal leave) OR (family planning) OR (family resource center) OR (community health center) OR (community land trust) OR (community revitalization) OR (community engagement) OR (community foundations) OR (community grant) OR (development screening) OR (green space OR park OR playground) OR (high-quality education) OR (housing policy) OR (housing subsidy) OR (inclusionary zoning) OR (justice) OR (labor market) OR (law enforcement OR police contact) OR (job training OR career training OR technical education OR technical school OR job corps OR trade school) OR (waiting period) OR (work schedule OR consistent work schedule OR commute OR work from home OR flexible work schedule) OR (livable wage) OR (youth program) OR (public housing OR subsidized housing) OR (public safety) OR (public transport OR transit OR reliable transport) OR (reproductive health care) OR (restorative justice) OR (safe housing) OR (perinatal care OR prenatal) OR (behavioral health OR mental health) OR (civic engagement) OR (sex education OR comprehensive sex education) OR (SNAP OR food stamps OR CalFresh) OR (Social and Emotional Learning OR SEL OR Social Emotional Learning) OR (social norm) OR (sense of belonging OR community integration OR neighbor cohesion OR community cohesion) OR (social capital) OR (social control) OR (social integration OR social cohesion) OR (state hospital OR local hospital) OR (TANF OR Temporary Assistance for Needy Families) OR (computer access OR internet access OR smartphone OR computer OR internet) OR (telehealth) OR (urban design OR urban plan OR municipal plan OR land use OR land use policy) OR (voter turnout) (collective impact OR cross-sector partner) OR (community action partnership) OR (two generation approach OR 2-gen approach OR inter-generation OR multi-generation) OR (black OR Asian OR Native American OR American Indian OR Hispanic OR latin OR white OR Pacific Islander OR POC OR people of color OR BIPOC) OR (immigrant status OR citizen) OR (LGBT OR LGB OR sexual orientation OR gender identity OR queer) OR (housing cost burden OR housing cost OR housing affordability) OR (environmental contaminant OR environmental hazard) OR (vacant housing))
AND Terms related to child well-being	((child OR toddler OR infant OR baby OR school age child)) AND ((well-being OR development OR health OR outcome) OR (Adverse Childhood Experience OR ACEs) OR (attachment OR attachment style) OR (crime) OR (discrimination) OR (food insecurity) OR (frequent moving OR displacement) OR (gender inequality) OR (gentrification) OR (gun violence OR firearm OR gun) OR (community violence OR neighborhood violence) OR (harsh parenting) OR (high school graduation) OR (homeless OR houseless OR homeowner) OR (housing insecurity) OR (incarceration of a family member OR family member in jail OR family in jail OR incarceration of family OR family in prison) OR (income inequality) OR (intimate partner violence OR partner violence OR domestic violence) OR (literacy OR reading level) OR (low income OR poverty OR child poverty) OR (median income) OR (parent substance abuse) OR (parent-child relationship) OR (poverty line) OR (residential stability OR housing stability) OR (segregation) OR (single parent) OR (systemic racism) OR (teen pregnancy) OR (social competence OR emotional competence) OR (toxic substance) OR (trauma) OR (unemployment) OR (verbal threat OR physical threat OR criticize OR verbal abuse OR physical abuse OR emotional abuse OR neglect OR mental violence OR emotional maltreatment) OR (violent crime))
And Language selection	“English” [Language]
AND Date of publication	1 January 2013–31 November 2023

## Supplementary Materials

The following supporting information can be downloaded at https://www.mdpi.com/article/10.3390/children11091070/s1, Prisma Checklist S1. Reference [367] is cited in the Supplementary Materials.
